# High-Performance Barium Titanate, Carbon Nanotube, and Styrene–Butadiene Rubber-Based Single Composite TENG for Energy Harvesting and Handwriting Recognition

**DOI:** 10.3390/polym17152016

**Published:** 2025-07-23

**Authors:** Md Najib Alam, Vineet Kumar, Youjung Kim, Dong-Joo Lee, Sang-Shin Park

**Affiliations:** School of Mechanical Engineering, Yeungnam University, 280, Daehak-ro, Gyeongsan 38541, Republic of Korea; mdnajib.alam3@gmail.com (M.N.A.); vineetfri@gmail.com (V.K.); msyzlmc@naver.com (Y.K.); djlee@yu.ac.kr (D.-J.L.)

**Keywords:** triboelectric nanogenerator, barium titanate, carbon nanotubes, rubber nanocomposite, energy harvesting, handwriting recognition

## Abstract

In this research, a single composite-type stretchable triboelectric nanogenerator (TENG) is proposed for efficient energy harvesting and handwriting recognition. The composite TENGs were fabricated by blending dielectric barium titanate (BT) and conductive carbon nanotubes (CNTs) in varying amounts into a styrene–butadiene rubber matrix. The energy harvesting efficiency depends on the type and amount of fillers, as well as their dispersion within the matrix. Stearic acid modification of BT enables near-nanoscale filler distribution, resulting in high energy conversion efficiencies. The composite achieved power efficiency, power density, charge efficiency, and charge density values of 1.127 nW/N, 8.258 mW/m^3^, 0.146 nC/N, and 1.072 mC/m^3^, respectively, under only 2% cyclic compressive strain at 0.85 Hz. The material performs better at low stress–strain ranges, exhibiting higher charge efficiency. The generated charge in the TENG composite is well correlated with the compressive stress, which provides a minimum activation pressure of 0.144 kPa, making it suitable for low-pressure sensing applications. A flat composite with dimensions of 0.02 × 6 × 5 cm^3^ can produce a power density of 26.04 W/m^3^, a charge density of 0.205 mC/m^3^, and an output voltage of 10 V from a single hand pat. The rubber composite also demonstrates high accuracy in handwriting recognition across different individuals, with clear differences in sensitivity curves. Repeated attempts by the same person show minimal deviation (<5%) in writing time. Additionally, the presence of reinforcing fillers enhances mechanical strength and durability, making the composite suitable for long-term cyclic energy harvesting and wearable sensor applications.

## 1. Introduction

Electrical energy derived from physical processes has become increasingly viable due to advancements in materials and technology. Although the energy produced through these processes is comparatively low, it does not have the harmful environmental impacts associated with chemical processes. Recently, nanogenerators have been developed to generate energy on both small and large scales from mechanical motion or temperature gradients [1]. Among various types of nanogenerators, triboelectric nanogenerators (TENGs) have gained significant attention for their ability to harvest energy from mechanical motion [2]. However, several challenges remain, including low power density, material degradation due to friction, low stretchability, environmental sensitivity, limitations in large-scale production, irregular output, charge retention issues, fabrication complexity, and compatibility with existing systems [3,4,5,6,7,8]. Overcoming these challenges could enable TENGs to provide scalable energy solutions that can be utilized across a wide range of intelligent and commercial applications [9,10,11,12,13,14].

Material selection and fabrication techniques are crucial for achieving high-power TENGs. Ideally, the selected materials should possess high dielectric constants, wide electron affinity differences, and large surface areas [15,16,17,18]. Since TENGs rely on contact and separation mechanisms to generate electrical energy, significant physical interaction between the interfaces is essential. This interaction leads to higher frictional forces, which, in turn, generate more triboelectric charges [19,20]. The materials used in triboelectric nanogenerators are diverse, including metals as conductors, dielectric polymers, and inorganic materials [21]. Several parameters must be considered for practical applications, including power efficiency, stretchability, mechanical stability, and material compatibility.

Among various electrically conductive materials, carbon nanotubes (CNTs) have emerged as attractive components in TENGs. Okochi et al. [22] found that carbon nanotube composite papers can serve as TENGs with promising output voltages. In another study, Kınas et al. [23] demonstrated that a 3 wt% CNT-based polyacrylonitrile nanofiber TENG composite (4 × 4 cm^2^) could generate 960 V and had a charge capacity of 260 nC under a 14.6 MΩ load resistance and a 0.022 µF capacitive load, respectively. Wang et al. [24] developed a TENG incorporating 0.5% CNTs into a supramolecular polyrotaxane matrix, achieving a power density of 385 mW/m^2^ at a 20 MΩ load resistance. Although polymer-based composites [22,23,24] show significant output power densities, they may exhibit limitations in mechanical properties such as stretchability. To address this, Lee et al. [25] developed a poly (dimethylsiloxane) (PDMS)-based TENG using surface-modified CNTs via pulsed laser ablation, achieving excellent power density. Similarly, Matsunaga et al. [26] demonstrated a transparent and stretchable TENG fabricated with a thin CNT film over a PDMS substrate, achieving excellent power output suitable for soft wearable applications.

Dielectric ceramic materials play a significant role in enhancing the dielectric properties of TENG components, thereby improving charge accumulation and polarization, which leads to higher energy harvesting efficiencies [17,27,28,29]. Barium titanate, a ceramic material with a high dielectric constant and excellent binding capability with polymers, is an attractive choice for TENG composites [30,31,32,33,34]. Pandey et al. [30] fabricated a Nafion-functionalized barium titanate and polyvinylidene fluoride composite, which demonstrated several times higher output voltage, current density, and power density compared to pristine polyvinylidene fluoride. Sun et al. [31] achieved a high-performance TENG by using aminated barium titanate and functionalized graphite oxide with polyimide as a substrate, aimed at the early detection of Parkinson’s disease. Yan et al. [32] demonstrated a TENG with a polyimide nanofiber and a piezoelectric carbon nanotube/barium titanate hybrid filler-based composite, resulting in three times higher energy output compared to pristine polyimide. They also achieved a maximum power density of 5.3 W/m^2^ at a 5 MΩ loading resistance and a maximum charge of approximately 80 nC with 25 N of applied force. Shuai-Bo et al. [33] developed a paper-based TENG using barium titanate and bamboo cellulose, achieving 13.51 µA of current, 118.5 V output voltage, and a maximum power density of 0.36 W/m^2^ at a 5 MΩ load resistance, significantly outperforming pure cellulose paper. Similarly, Patil et al. [34] achieved 103 V output voltage, 3.6 µA current, and a maximum power density of 32 µW/cm^2^ at 40 N of applied force with a copy paper and barium titanate-based composite. From this discussion, it is evident that TENG performance varies significantly with material properties, and thus, the choice of materials is critical for achieving higher energy conversion efficiency.

Recently, rubber has emerged as a viable candidate for flexible and stretchable TENG applications [35,36,37,38,39]. As a tribo-negative material, rubber can be combined with tribo-positive materials to enhance TENG efficiency. Unlike other polymeric materials, rubber offers a wide elasticity range and robust stretchability. Additionally, due to stronger physical and chemical interactions with various fillers, the mechanical properties of rubber-based composites can be significantly improved. As a result, rubber composite-based TENGs are suitable for use in durable electronic and energy-harvesting systems.

Appamato et al. [35] fabricated natural rubber-Ag nanocomposites with different surfactants, finding that cationic surfactant CTAB-capped Ag nanoparticles resulted in higher power densities compared to anionic SDS-modified composites, due to better filler dispersion and dielectric enhancements. These composites achieved a maximum output voltage of 120 V and a power density of 0.83 W/m^2^, which is six times higher than unfilled natural rubber. The composites also exhibited antibacterial activity and demonstrated energy harvesting from walking using a TENG film integrated into a shoe insole. In another study, Chomjun et al. [36] obtained a maximum power density of 0.242 W/m^2^ and an output voltage of 89.6 V using a natural rubber and activated carbon composite. Candido et al. [37] fabricated all-silicone rubber-based TENGs, achieving a maximum power density of 197.2 µW/cm^2^ and an output voltage of 308.7 V with graphite-impregnated electrodes. Lu et al. [38] developed a TENG with carboxyl-functionalized nitrile butadiene rubber, achieving a maximum power density of 6.14 W/m^2^. Gao et al. [39] created a TENG from a special rubber prepared by grafting acrylamide and hydroxyethyl acrylate to carboxylated nitrile butadiene rubber, obtaining an open-circuit voltage of 723 V and a power density of 3.25 W/m^2^. These results highlight that output voltage, current, and power density vary with different rubber composites, likely due to differences in electron affinity values and measurement techniques. A TENG composite with higher resistivity may produce higher peak voltage and current according to Ohm’s law. Similarly, maximum power density can be achieved at an optimum loading resistance. However, charge generation primarily depends on the electron affinity differences and the interfacial areas of interaction between the materials. Therefore, for high-performance TENGs, the composite should exhibit a high electron affinity difference and large interfacial areas of interaction.

Although the separated triboelectric interfaces in a TENG provide better peak voltages due to the non-conducting nature of the films, the peaks are often non-symmetric and irregular with mechanical loads. This could be attributed to the non-homogeneous mechanical load transfer caused by distinctly separated triboelectric interfaces in the two-electrode-based TENG system, which limits its application as a physical sensor. Another disadvantage of the two-electrode TENG system is the lower mechanical stability when sufficiently thin films are used to enhance performance. In contrast, a single-electrode type TENG produces lower output currents and voltages but exhibits more symmetric responses to mechanical loads due to the homogeneous distribution of the triboelectric interfaces in the composite. Considering these challenges, we have proposed a single-electrode type TENG fabricated by incorporating conducting carbon nanotubes (CNTs) and high-dielectric barium titanate (BT) into a styrene–butadiene rubber (SBR) matrix. SBR is a synthetic rubber known for its high industrial value due to its elasticity, stretchability, abrasion resistance, and ability to be reinforced with various fillers through physical and chemical interactions [40,41,42,43]. Moreover, SBR contains a large number of benzene rings as π-electron systems, which strongly interact with carbon materials such as carbon nanotubes through π–π interactions. This significantly improves the mechanical, electrical, and electromechanical properties of the rubber composite [43]. Unlike other diene rubbers, SBR also has a lower number of reactive unsaturations in its molecular chains, providing better aging resistance [44] and making it more suitable for harsh environments. Furthermore, SBR belongs to the tribo-negative side, while barium titanate belongs to the tribo-positive side in the triboelectric series, facilitating higher triboelectric charge generation. Given these properties, SBR and the selected fillers were deliberately chosen to formulate a new TENG composite aimed at achieving higher performance.

Here, we propose a single-electrode TENG composite, where electrically conducting networks in the rubber matrix act as the electrode, and rubber and barium titanate interfaces serve as triboelectric layers, forming numerous hypothetical triboelectric cells within the rubber composite. Upon the application of mechanical energy, even at very low deformation, these cells generate a significant amount of triboelectric charge due to internal friction between the rubber and barium titanate particles. This TENG offers several advantages, including symmetric output voltage and current, possibly due to the homogeneous distribution of the filler, which results in smooth mechanical load transfer. Moreover, due to the strong reinforcing abilities of the filler materials, the composite becomes more robust and gains higher mechanical stability, which is essential for a durable energy-harvesting system. To understand the triboelectric behaviors of different rubber composites with varying filler amounts, power efficiency, power density, charge efficiency, and charge density were measured. In a triboelectric cell, both the interactive area and the electrode area are critical factors; thus, optimal conductivity and an optimal filler–rubber interfacial area can result in the highest triboelectric charge generation. To investigate the triboelectric interactions between barium titanate and the rubber phases, barium titanate was modified with stearic acid, with the belief that, at an optimal concentration, it would enhance filler dispersion in the rubber matrix, thereby resulting in higher triboelectric charge generation. Beyond the optimum concentration, excess unreacted stearic acid may remain between the triboelectric layers, reducing triboelectric efficiencies. The results support this hypothesis. Finally, the optimum TENG composite was demonstrated for longer energy-harvesting cycles and personal handwriting recognition.

## 2. Materials and Methods

### 2.1. Materials

**Figure 1:**
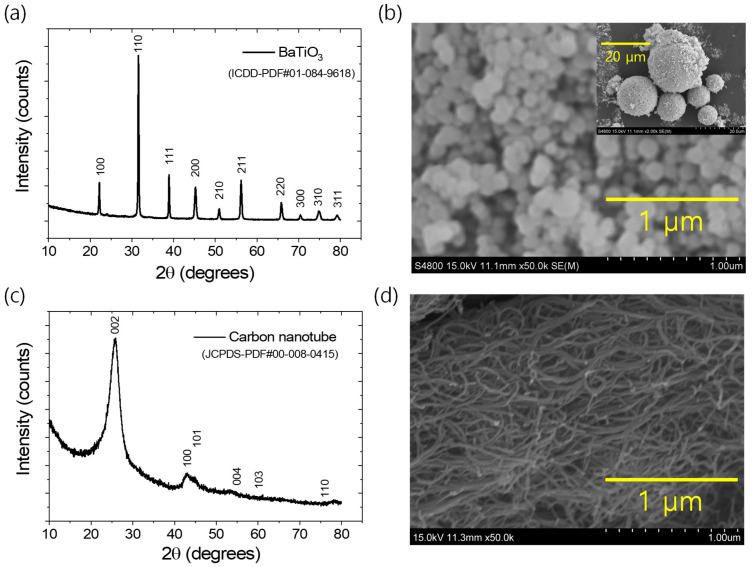
(**a**) XRD plot BaTiO_3_, (**b**) SEM image images of BaTiO_3_, (**c**) XRD plot of CNT, and (**d**) SEM image of CNT.

Styrene–butadiene rubber (SBR-1502, approximately 23.5% styrene monomer content) was procured from Kumho Petrochemicals, Seoul, Republic of Korea. A masterbatch of unvulcanized rubber was formulated using a two-roll mixing mill, incorporating curing ingredients such as zinc oxide, stearic acid, tetramethyl thiuram disulfide, N-tert-butyl-benzothiazole sulfonamide, and sulfur in the amounts of 5, 2, 1, 1.75, and 1.5 g, respectively, per 100 g of raw rubber, as described elsewhere [42,43]. Barium titanate (BT), with a primary particle size of 100 nm, was purchased from US Research Nanomaterials, Inc., Houston, TX, USA. Multiwalled carbon nanotubes (CNT, CNT-100), with a specific surface area of 250 m^2^/g, were purchased from Hanwha Nanotech Corporation Ltd., Seoul, Republic of Korea. Both fillers were further characterized by X-ray diffraction (XRD, PANalytical XpertPro, Malvern, Worcestershire, UK) and scanning electron microscopy (SEM, S-4800, Hitachi, Tokyo, Japan) to examine their crystal structures and morphology. The results are provided in Figure 1a–d. Figure 1a shows the cubic crystalline structure of barium titanate, according to the ICDD-PDF#01-084-9618 [45]. The SEM image in Figure 1b indicates that the primary particle dimensions range within 100 nm, although the agglomerated filler structures vary from a few micrometers to several micrometers (as shown in the inset of Figure 1b). The XRD result in Figure 1c confirms that CNTs exhibit a hexagonal graphitic crystal structure, according to the reference JCPDS PDF#00-008-0415. The SEM image reveals that the CNTs have tubular morphologies, with individual tube diameters of less than 50 nm and lengths extending to a few micrometers.

### 2.2. Fabrication of Rubber Composites

Before preparing the vulcanized rubber composites, the filler materials were mixed with the unvulcanized masterbatch rubber using a solvent blending technique. This method offers several advantages, including uniform filler dispersion, efficient wetting of fillers by rubber chains, and enhanced electrical properties in the resulting rubber composites. Additionally, it minimizes environmental impact when the solvent is used in a controlled manner, as described previously [42,43,46].

First, 25 g of masterbatch rubber was taken in a glass jar, and 80 mL of toluene was added and left to soak for about 18–20 h. After soaking, the rubber was converted into a slurry by mechanical stirring. In another glass container, the requisite amount of barium titanate from Table 1 was dispersed in 100 mL of toluene and sonicated for 1 h. The container was then placed in an oven at 100 °C to evaporate the solvent, reducing the total volume by half. For stearic acid-modified barium titanate-filled composites, the required amount of stearic acid from Table 1 was first dissolved in toluene before adding the barium titanate, followed by sonication. After removing the barium titanate solution from the oven, the requisite amount of CNTs was added to the solution, and the entire mixture was further sonicated for another 30 min. In the next step, the rubber slurry and the solvent-blended fillers were mixed by vigorous mechanical stirring for about 10 min. The final slurry was then placed in a flat tray and dried in an oven at 80 °C for about 24 h. The dried rubber compounds were subsequently cured (vulcanized) into sheet and cylindrical shapes, as presented in Figure S1a (Supporting Information), using a hot press molding machine at 150 °C for 15 min [42,43].

### 2.3. Measurements of Mechanical Properties

Mechanical properties, including compressive and tensile behavior, were investigated using a Universal Testing Machine (Lloyd UTM, Westminster, UK) equipped with a 1 kN load cell. For compressive tests, cylindrical samples (height = 10 mm, diameter = 20 mm) were compressed up to 20% strain at a motor speed of 2 mm/min. For tensile tests, dumbbell-shaped specimens (ISO-37 [47], Type-2) were used, with the motor speed set to 300 mm/min. To determine the average value of each specific property, four consecutive tests were conducted.

### 2.4. Filler Dispersion of Rubber Composites

The dispersion of fillers in the rubber matrix was studied using the aforementioned SEM instrument. Tensile fracture surfaces of the rubber composites were used for SEM analysis. Prior to imaging, the samples were sputter-coated with gold.

### 2.5. Measurements of Triboelectric Properties

A digital multimeter (Keysight 34461A, Keysight, Santa Rosa, CA, USA) and a loading machine (UTM) were used to measure the triboelectric performance in terms of power efficiency, power density, charge efficiency, and charge density under a 2% compressive loading–unloading cycle of cylindrical specimens (height = 10 mm, diameter = 20 mm). These parameters were calculated using the following equations (Equations (1)–(4)) at a frequency of 0.85 Hz, based on the average of ten cycles:

(1) $$Power\;efficiency=\frac{\Delta I\cdot \Delta V}{N}$$

(2) $$Power\;density=\frac{\Delta I\cdot \Delta V}{V_{n}}$$

(3) $$Charge\;efficiency=\frac{\int_{0}^{t}I\left(t\right)\mathrm{dt}}{N}$$

(4) $$Charge\;density=\frac{\int_{0}^{t}I\left(t\right)\mathrm{dt}}{V_{n}}$$

where ΔI is the difference between the maximum and minimum current values, ΔV is the difference between the maximum and minimum voltage values, *N* is the applied force, *Vₙ* is the volume of the cylindrical sample, and I(t) is the time-dependent current.

It is well known that electrical charge is the product of current and time, which corresponds to the area under the current–time curve. In this case, the total electrical charge per cycle is obtained by integrating both the negative and positive areas under the current–time plot over the full cycle duration from 0 to t.

Figure S1b illustrates the components of the triboelectric nanogenerator (TENG), while Figure S1c,d show the experimental setup with the actual sample.

## 3. Results and Discussion

### 3.1. Mechanical Properties of the Rubber Composites

**Figure 2:**
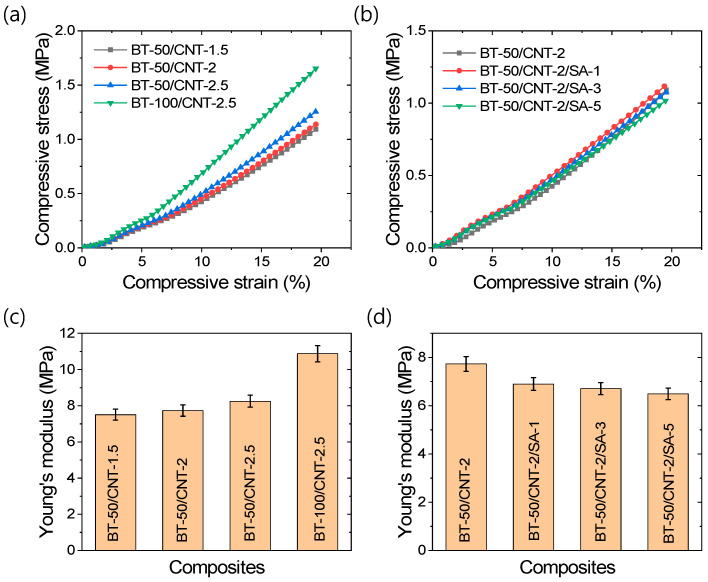
Compressive mechanical properties of rubber composites; (**a**,**b**) Stress–strain curves, and (**c**,**d**) Young’s modulus.

Mechanical properties are critical parameters for ensuring stable TENG applications. Therefore, it is important to describe the mechanical behavior of the rubber composites before discussing their TENG performance. Various compressive mechanical behaviors are illustrated in Figure 2a–d. The compressive stress–strain curves in Figure 2a,b indicate that compressive stress increases with increasing strain. As shown in Figure 2a, increasing the amount of carbon nanotubes (CNTs) or barium titanate (BT) results in steeper stress–strain slopes, suggesting enhanced stiffness. In contrast, Figure 2b shows that, at a constant BT content, increasing the amount of stearic acid modifiers does not significantly affect the slope. From the Young’s modulus values presented in Figure 2c,d, it can be observed that the addition of CNTs or BT increases the modulus, while the inclusion of stearic acid as a filler modifier decreases the modulus. This indicates that CNTs and BT act as reinforcing fillers, whereas stearic acid reduces the reinforcing capability of the filler materials. Several interactions contribute to the enhancement of compressive modulus in filled rubber composites [48]. Among them, occluded rubber chains within the filler structures significantly reduce the effective volume of soft rubber domains, and the higher stress transfer through rigid filler networks results in increased stiffness or Young’s modulus. On the other hand, stearic acid promotes the deagglomeration of filler particles into primary particles, thereby reducing the effectiveness of stress transfer and leading to decreased stiffness or modulus values compared to unmodified composites. This observation is supported by the SEM studies discussed in later sections. Interestingly, although at the lowest amount of stearic acid the Young’s modulus is lower than that of the unmodified composite, the modified composite maintains a similar modulus, as observed from the stress–strain curves in Figure 2b. This may be attributed to improved filler–polymer interactions, possibly due to the increased effective surface area of the BT particles in the rubber composites following filler modification [48,49]. For easier reference of load values corresponding to different strain levels, Figure S2a,b are provided in the Supporting Information section.

**Figure 3:**
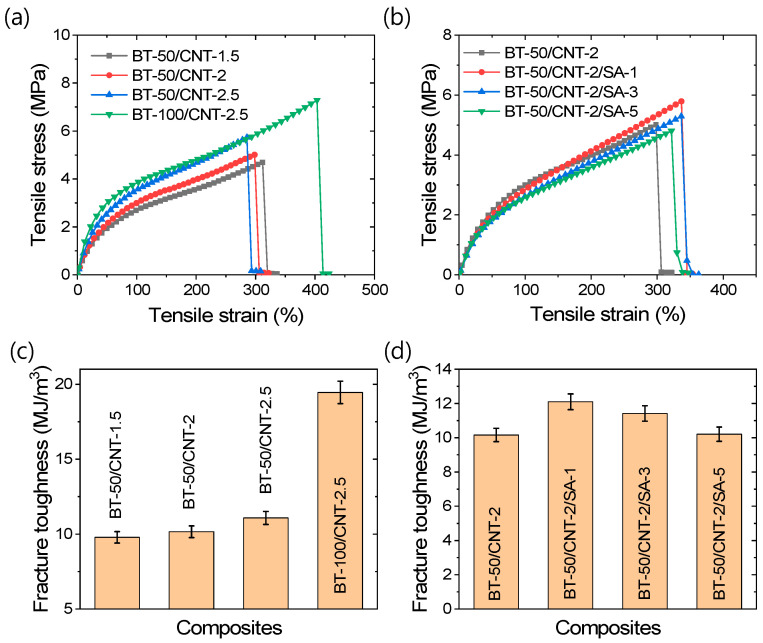
Tensile mechanical properties of rubber composites; (**a**,**b**) stress–strain curves and (**c**,**d**) fracture toughness values.

Tensile mechanical properties are important for the stretchable applications of rubber composites. It has been reported that CNT-based rubber composites exhibit excellent strain sensitivity [43,50,51]. For high-performance strain sensors, mechanical stability and stretchability are crucial parameters, along with linearity in strain sensitivity [43,50,51]. Various tensile properties of the rubber composites are presented in Figure 3a–d. From the stress–strain curves in Figure 3a, it is evident that increasing the filler content enhances the tensile strength. Unlike CNT, increasing the amount of BT leads to an improvement in the elongation at break. Figure 3b further shows that the addition of stearic acid, up to an optimal amount, results in increased tensile strength as well as elongation at break. The stress–strain curves in Figure 3b also reveal smoother profiles for stearic acid-modified composites, which can be attributed to improved filler distribution within the rubber matrix. Fracture toughness, which is directly related to the mechanical stability of rubber composites, is shown in Figure 3c,d. These figures indicate that fracture toughness improves with increasing filler content, with BT-rich composites demonstrating particularly high values. Since fracture toughness is influenced by both tensile strength and elongation at break, a significant increase in elongation can contribute to enhanced toughness. As shown in Figure 3d, stearic acid modification of the filler significantly increases the fracture toughness and overall stability of the composites. Due to the smooth stress–strain response and improved mechanical stability, the stearic acid-modified composite demonstrates excellent potential for strain sensing applications, exhibiting a fracture toughness of 12.1 MJ/m^3^ and an elongation at break of approximately 337%, which surpass previously reported values for similar CNT content in SBR composites [43]. The variation in tensile load with strain is provided in Figure S3a,b in the Supporting Information section.

**Figure 4:**
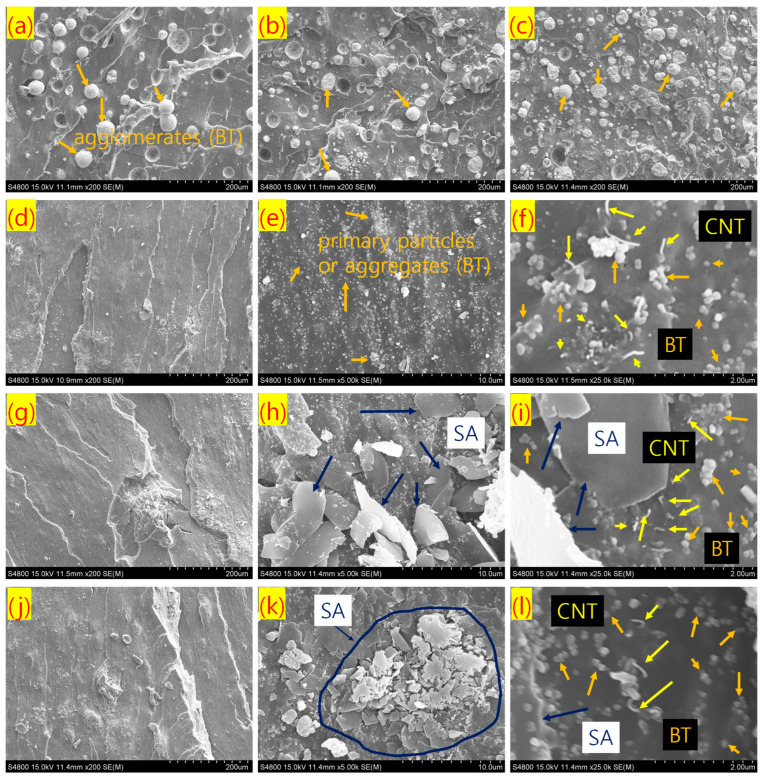
SEM images of tensile fractured rubber composites with different magnification; (**a**) BT-50/CNT-2 at 200×, (**b**) BT-50/CNT-2.5 at 200×, (**c**) BT-100/CNT-2.5 at 200×, (**d**) BT-50/CNT-2/SA-1 at 200×, (**e**) BT-50/CNT-2/SA-1 at 5.0k×, (**f**) BT-50/CNT-2/SA-1 at 25.0k×, (**g**) BT-50/CNT-2/SA-3 at 200×, (**h**) BT-50/CNT-2/SA-3 at 5.0k×, (**i**) BT-50/CNT-2/SA-3 at 25.0k×, (**j**) BT-50/CNT-2/SA-5 at 200×, (**k**) BT-50/CNT-2/SA-5 at 5.0k×, and (**l**) BT-50/CNT-2/SA-5 at 25.0k×.

SEM images of different rubber composites are presented in Figure 4a–l. Figure 4a–c reveal that, without stearic acid modification, BT particles exhibit poor dispersibility in the rubber matrix, with many remaining in the form of agglomerated filler structures. However, increasing the filler content, whether CNT or BT, slightly improves filler distribution homogeneity, contributing to the enhanced mechanical properties observed with higher filler loading. When stearic acid is introduced as a filler modifier, a significantly improved dispersion of filler particles is achieved, with predominantly primary filler structures, as observed in Figure 4d–f. Specifically, Figure 4d shows no visible agglomeration of BT particles, unlike the structures seen in Figure 4a–c. In Figure 4e,f, both BT and CNT particles appear more uniformly dispersed throughout the matrix. This improved dispersion contributes to a marginal increase in fracture toughness when an optimal amount of stearic acid is used. Similarly, other stearic acid-modified composites also demonstrate excellent BT particle distribution, as seen in Figure 4g–l. However, when the stearic acid content exceeds 1 phr, the excess modifier—beyond the level required for optimal filler surface coverage—may deposit on the rubber matrix. Since unreacted stearic acid has low compatibility with the rubber, it does not further enhance fracture toughness and may even reduce it at higher concentrations. Although stearic acid substantially improves BT filler dispersion, no significant increase in Young’s modulus is observed. This suggests that the Young’s modulus is primarily governed by the integrity of the filler network structures [48]. In stearic acid-modified composites, these networks are largely broken down, resulting in reduced modulus values. However, the more homogeneous, nanoscale distribution of fillers in these modified composites can enhance elongation properties. As there is no notable improvement in tensile strength following filler modification, it can be inferred that BT particles have relatively weak physical interactions with rubber chains, possibly due to a large polarity mismatch. Overall, stearic acid modification results in softer composites with enhanced elongation at break, which may lead to improved fracture toughness compared to unmodified systems.

### 3.2. Triboelectric Behaviors of the Rubber Composites

**Figure 5:**
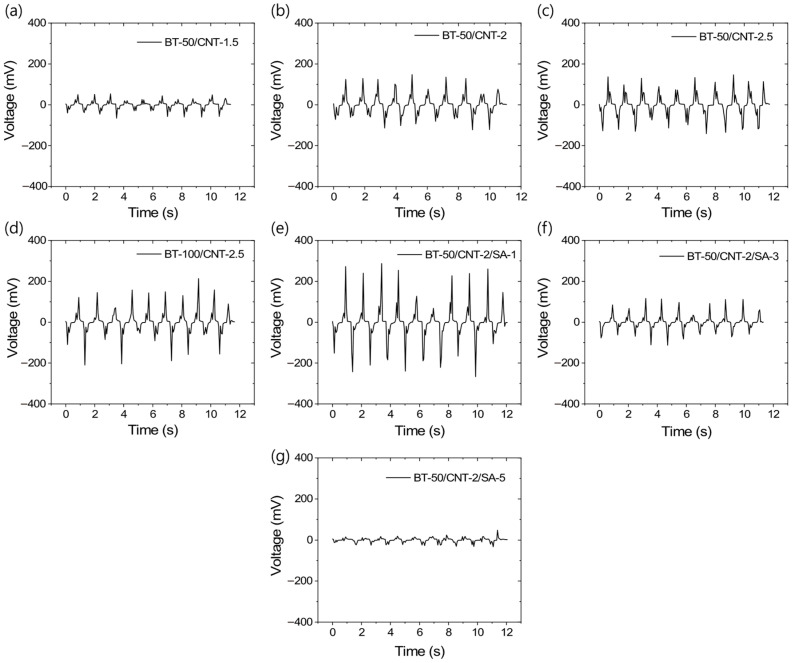
TENG output voltage profiles for different rubber composites at 2% dynamic strain and 10 loading–unloading cycles; (**a**) BT-50/CNT-1.5, (**b**) BT-50/CNT-2, (**c**) BT-50/CNT-2.5, (**d**) BT-100/CNT-2.5, (**e**) BT-50/CNT-2/SA-1, (**f**) BT-50/CNT-2/SA-3, and (**g**) BT-50/CNT-2/SA-5.

The output voltage generation behavior of the triboelectric rubber composites is presented in Figure 5a–g. Figure 5a–c demonstrate that the output voltage significantly increases with increasing CNT content from 1.5 to 2 phr. However, beyond 2 phr CNT, only a marginal improvement in voltage output is observed. Similarly, at a fixed CNT content, increasing the BT concentration further enhances the output voltage. This indicates that the composite-based TENG achieves maximum output voltage when both the conductive network formed by CNTs and the interfacial interaction area between rubber and BT particles are optimized. At low conductivity, a portion of the generated charge may not be effectively extracted, while at high conductivity continuous conductive pathways may form between the rubber and BT particles, reducing triboelectric charge generation or enhancing charge relaxation due to direct connections between the triboelectric layers by conducting CNTs. Interestingly, when the BT content is doubled at a fixed CNT level, no significant improvement in output voltage is observed compared to the composite with lower BT content (Figure 5d). This initially suggested poor BT filler dispersion, which was later confirmed by SEM analysis. SEM studies revealed that the dispersion of BT fillers can be significantly enhanced by modifying them with stearic acid. The output voltages of the composites containing stearic acid-modified fillers are shown in Figure 5e–g. Notably, up to an optimal amount, stearic acid modification improves both filler dispersion and output voltage. However, exceeding the optimal quantity results in a decline in output voltage, despite maintaining good filler dispersion. It is believed that, at lower concentrations, stearic acid efficiently modifies the BT particle surfaces, reducing particle size and weakening strong filler–filler interactions, while any excess stearic acid may dissolve in the rubber matrix. This results in a larger triboelectric surface area and the highest output voltage, as seen in Figure 5e. Beyond the optimal level, excess stearic acid may remain undissolved and precipitate within the matrix, as observed in SEM images. The presence of this layer between the triboelectric interfaces can hinder effective charge generation, leading to reduced output voltage, as shown in Figure 5f,g. Another possible explanation is that higher levels of stearic acid cause over-coating of filler particles, which may reduce the electrical conductivity. Therefore, an optimal stearic acid content of 1 phr appears to provide the best balance between filler distribution and triboelectric voltage output.

**Figure 6:**
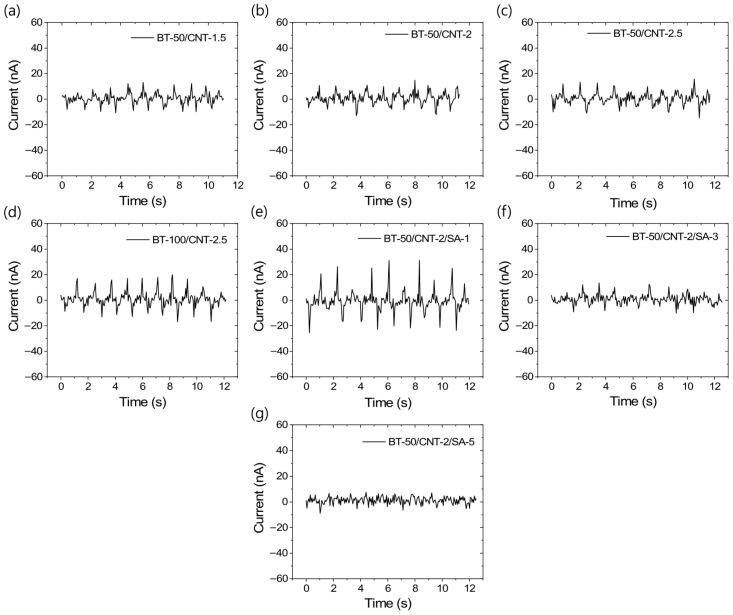
TENG output current profiles for different rubber composites at 2% dynamic strain and 10 loading–unloading cycles; (**a**) BT-50/CNT-1.5, (**b**) BT-50/CNT-2, (**c**) BT-50/CNT-2.5, (**d**) BT-100/CNT-2.5, (**e**) BT-50/CNT-2/SA-1, (**f**) BT-50/CNT-2/SA-3, and (**g**) BT-50/CNT-2/SA-5.

Similar to the voltage outputs, the output current values also follow the same trend, as shown in Figure 6a–g. These figures indicate that, at higher concentrations of stearic acid, the current outputs are significantly reduced, even falling below those of the unmodified composites. Figure S4a–d in the Supporting Information section present the average voltage and current responses under 2% compressive deformation cycles, which are useful for evaluating triboelectric efficiency. From these data, it is evident that the composite modified with 1 phr stearic acid (BT-50/CNT-2/SA-1) exhibits the best performance, achieving output values of approximately 500 mV and 50 nA under 2% compressive strain—significantly higher than all other composites tested.

**Figure 7:**
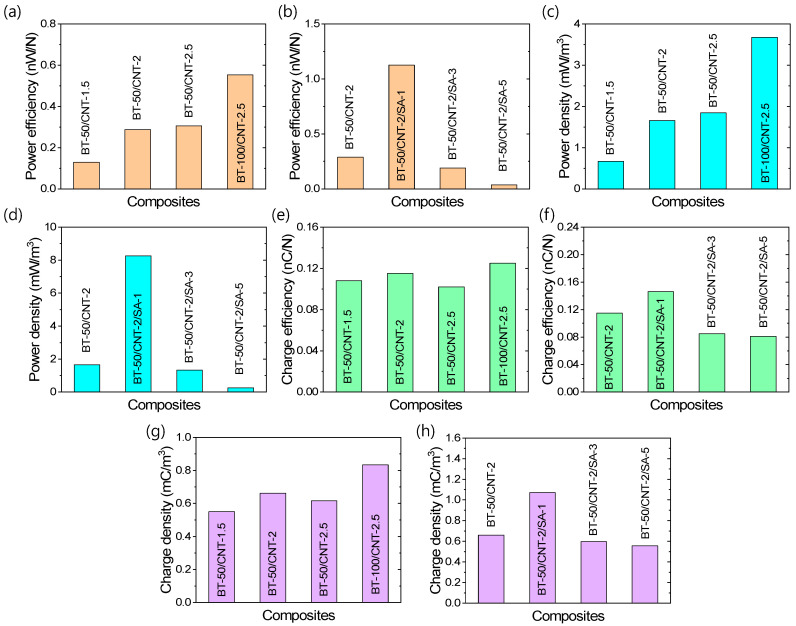
TENG efficiencies of the rubber composites under 2% compressive cyclic strain; (**a**,**b**) maximum power efficiency, (**c**,**d**) maximum power density, (**e**,**f**) charge efficiency, and (**g**,**h**) charge density.

Different types of TENG efficiencies are presented in Figure 7a–h. Regarding the maximum power efficiency and power density under a 2% deformation cycle, Figure 7a–d show that the addition of CNTs or BT leads to improvements in both parameters. Furthermore, improved filler dispersion—achieved with up to 1 phr of stearic acid—also enhances power efficiency and power density. However, at stearic acid contents above this optimum level, both metrics decline. The BT-50/CNT-2/SA-1 composite exhibits the highest performance, with a power efficiency of 1.127 nW/N and a power density of 8.258 mW/m^3^ at a frequency of 0.85 Hz under a 2% compressive loading–unloading cycle of the cylindrical sample. These values represent improvements of approximately 290% and 400%, respectively, compared to the corresponding unmodified composite (BT-50/CNT-2). Figure 7e–h illustrate the trends in charge efficiency and charge density. These figures reveal that increasing the CNT content up to an optimum of 2 phr enhances both charge efficiency and charge density. Beyond this point, further increases in CNTs reduce these values, likely due to the formation of continuous conductive networks between triboelectric interfaces, which promotes charge neutralization. This effect becomes clearer when BT content is increased at a fixed CNT level, as the enhanced triboelectric layer formation contributes to improved charge efficiencies. Since triboelectric efficiency is strongly influenced by the interfacial area between the filler and the rubber matrix, enhanced filler dispersion significantly boosts overall efficiency. The BT-50/CNT-2/SA-1 composite achieves a charge efficiency of 0.146 nC/N and a charge density of 1.072 mC/m^3^—approximately 27% and 62% higher, respectively, than those of the unmodified composite (BT-50/CNT-2). Although filler dispersion plays a critical role in enhancing TENG efficiencies, excessive stearic acid beyond the optimal amount reduces efficiency, likely due to the presence of a surface layer that interferes with effective triboelectric charge generation. Therefore, using a minimal, optimized amount of stearic acid is essential for achieving superior TENG performance.

**Figure 8:**
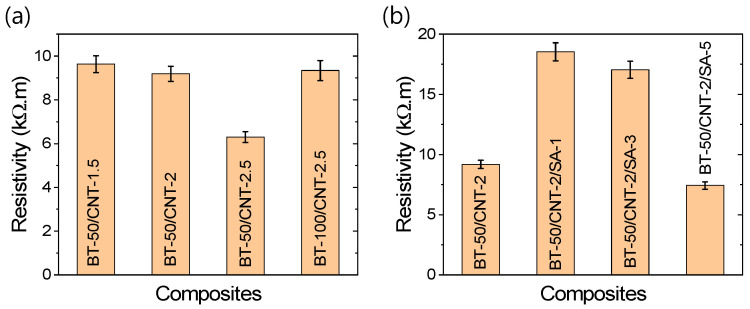
(**a**,**b**) Electrical resistivity of the rubber composites.

To explore potential correlations between resistivity and TENG performance, the resistivities of various composites were investigated, as shown in Figure 8a,b. These figures clearly demonstrate that the resistivity of the composites decreases with increasing CNT content, due to the formation of more conductive pathways. Conversely, increasing the amount of dielectric BT results in higher resistivity. At a fixed BT content, increasing the CNT content improves TENG performance, despite the associated reduction in resistivity. On the other hand, at a fixed CNT level, increasing the BT content enhances both TENG performance and resistivity. A similar trend is observed with improved filler dispersion—higher dispersion levels lead to enhanced TENG performance, even when the overall resistivity is higher compared to unmodified composites. These observations suggest that an optimal balance between the conductive (CNT) and dielectric (BT) components is essential for achieving high energy conversion efficiency in rubber-based TENGs. Specifically, a sufficient level of conductive network formation is necessary to support effective charge transport, while an appropriate amount of dielectric filler ensures efficient charge generation and storage.

**Figure 9:**
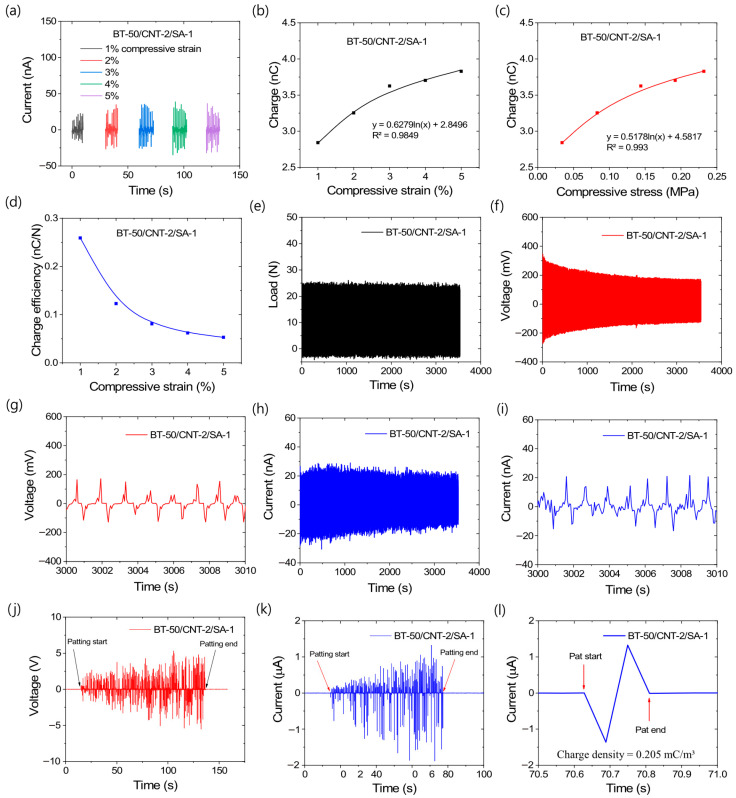
Triboelectric behavior of BT-50/CNT-2/SA-1 composite under different mechanical energy input systems; (**a**) current output with different cyclic compressive strains, (**b**) correlation of generated charge with compressive strains, (**c**) correlation of generated charge with compressive stress, (**d**) charge generation efficiencies under different compressive strain, (**e**) mechanical load profiles up to 3000 cycles, (**f**) voltage outputs up to 3000 cycles, (**g**) voltage outputs at higher cycles, (**h**) current outputs up to 3000 cycles, (**i**) current outputs at higher cycles, (**j**) voltage generation by patting, (**k**) current generation by patting, and (**l**) output current by a single pat.

Based on the above results and discussion, it is evident that the BT-50/CNT-2/SA-1 composite exhibits the best triboelectric performance among all studied formulations. Therefore, this composite was further evaluated for its energy harvesting behavior under various stress–strain conditions, extended cyclic loading–unloading tests, and biomechanical force applications. Figure 9a–l illustrate the energy harvesting characteristics of the BT-50/CNT-2/SA-1 composite under these different mechanical conditions. From Figure 9a–c, it can be observed that the amount of generated current or charge increases with increasing compressive strain and stress values. Figure 9a shows the current outputs at different cyclic strains ranging from 1% to 5%. The fitted curves in Figure 9b,c follow Arrhenius-type logarithmic equations, suggesting that triboelectric energy generation requires a minimum activation energy to initiate the energy conversion process. Since both mechanical deformation and pressure are related to the input mechanical energy, they exhibit clear correlations with the charge values through these equations. By extrapolating the curve to a charge value of zero, a minimum pressure of 0.144 kPa was obtained. Notably, the minimum pressure required to activate the BT-50/CNT-2/SA-1 composite is 0.144 kPa, which is considerably lower than common biomechanical pressures, such as systolic (16 kPa) and diastolic (11 kPa) blood pressures. This highlights the potential of the composite for future applications in physiological sensing, such as blood pressure monitoring. Interestingly, Figure 9d indicates that charge efficiencies are higher at lower strains, suggesting that applying a lower mechanical pressure is more efficient for energy conversion than a higher pressure. At elevated strain levels, the frictional interactions between the rubber matrix and BT particles are likely to increase, as is evident from the increased amount of generated charge. However, due to extended filler percolation, the generated charge may relax more rapidly as a result of increased conductivity. For this reason, the charge generation efficiency decreases at higher strains. These findings suggest that, rather than increasing the applied load on a thick TENG sample, expanding the surface area under a similar load could lead to enhanced energy output.

Figure 9e–i represent the energy harvesting behavior up to 3000 loading–unloading cycles with 2% applied strain in the cylindrical sample. From the load values in Figure 9e, there is no significant loss in mechanical load from the beginning to the end of the cycles. However, the output voltage shown in Figure 9f exhibits some degradation at higher cycle numbers. It can be seen from Figure S5a–d that, after approximately 2000 s of cyclic loading, there is no significant further decrease in the output voltage. From Figure 9g, it is clearly observed that, even at higher cycle times, the voltage output remains relatively stable. Similarly, Figure 9h,i show that the current output also remains stable at higher cycle times. The decrease in output voltage or current at higher cycles is believed to be due to the increase in the temperature of the sample caused by internal friction between different materials, particularly the friction between similar filler particles. Such friction does not produce triboelectric charges due to the absence of electronic affinity gaps but generates heat within the TENG. Although the resulting temperature increase is relatively low and does not significantly affect the bulk mechanical properties, it can have a notable effect on the charge generation efficiency depending on the temperature sensitivity of the TENG composite [52]. Although the durability was tested up to 3000 cycles, the degradation trends in output voltage and current suggest that the TENG composite can continue harvesting energy for many more cycles while maintaining similar efficiency, as observed after 2000 cycles.

As concluded previously, a thin TENG composite is expected to have higher energy conversion efficiency; therefore, a 0.02 cm-thick composite with an area of 6 × 5 cm^2^ was investigated for energy harvesting through patting. The voltage and current generation behaviors under biomechanical force are shown in Figure 9j–l. From Figure 9j,l, it can be seen that the voltage and current outputs reach approximately 10 V and 2 µA, respectively. Consequently, the power density for this sample can reach as high as 26.04 W/m^3^. Figure 9l shows that the calculated charge density for a single hand pat is as high as 0.205 mC/m^3^. The power density achieved by hand patting is comparatively better than other rubber-based TENG composites [35,36,37,38,39], despite their higher voltage outputs. Previous studies on different rubber composites [35,36,37,38,39] reported high triboelectric voltages, but the corresponding power densities were not significantly high relative to the high voltage values. Since power is the product of current and voltage, these earlier results indicate lower current densities or reduced charge generation. This behavior may be attributed to the sensitivity of the load measurement systems used, which can capture current or voltage with high temporal resolution. As total charge or energy output depends on both current and the duration of flow, higher power efficiencies do not necessarily correspond to higher charge efficiencies. For example, Appamato et al. [35] obtained a maximum power density of 0.83 W/m^2^ and a maximum output voltage of 120 V in natural rubber–Ag nanocomposites. The calculated current density was 6.92 mA/m^2^, which is much lower than that of the BT-50/CNT-2/SA-1 composite (26.04 mA/m^2^). Similarly, Gao et al. [39] reported a maximum voltage of 723 V and a power density of 3.25 W/m^2^, corresponding to a current density of 4.5 mA/m^2^. These data indicate that triboelectric charge extraction is more efficient in the BT-50/CNT-2/SA-1 composite due to its low resistivity or high conductivity. Moreover, the composite exhibits high stretchability and excellent fracture toughness. Considering all these factors, the BT-50/CNT-2/SA-1 composite demonstrates strong potential for effective energy harvesting from various forms of mechanical energy.

**Figure 10:**
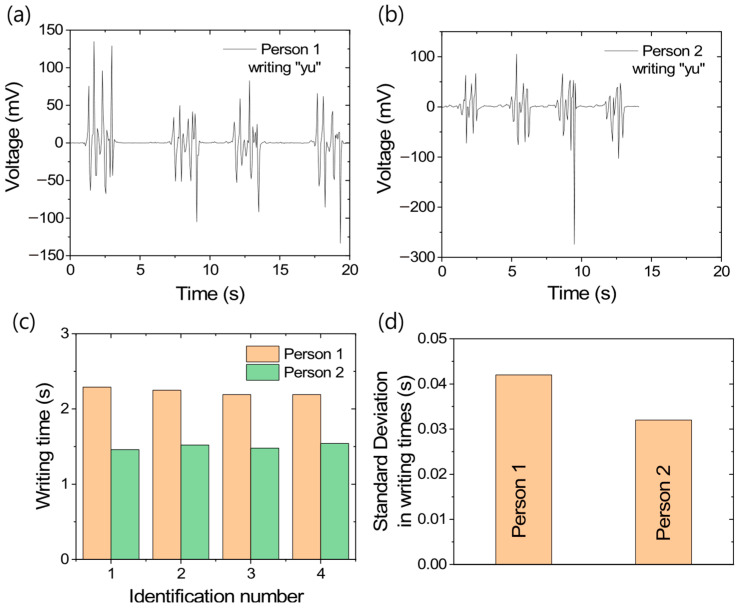
Handwriting recognition through TENG-based sensor; (**a**) output voltage patterns for Person 1, (**b**) output voltage patterns for Person 2, (**c**) writing times with different identification numbers, and (**d**) standard deviation in individual handwriting times.

Since the TENG composite exhibits highly symmetric positive and negative output voltages under mechanical loading, it was further tested for personal handwriting recognition. The voltage patterns generated while writing “yu” by different individuals are shown in Figure 10a,b. From these figures, it is evident that, for the same person, the voltage patterns are similar in nature; however, for different individuals, the patterns are distinctly different. In addition to the visual distinction, the time taken to write the letters also varies significantly, with differences exceeding 500 ms, as shown in Figure 10c. Although the writing times differ considerably, it is interesting to note that the personal errors remain below 5%, as presented in Figure 10d. In a similar study, Alam et al. [53] showed that writing a single number or character produces a similar output voltage pattern if written in the same direction or style. However, when the same symbol is written in the other direction or style, the voltage patterns become significantly different due to changes in timing and force application. Generally, while there is only a small difference in time and pattern when writing a single character, this difference tends to increase progressively from a charecter to a word and then to a sentence. While visually similar signatures can be replicated through simple writing, replicating a signature while maintaining the same writing duration is extremely difficult. Based on this concept, the TENG composite shows great potential for future applications in handwriting recognition.

## 4. Conclusions

In this research, single composite-type TENGs, where dispersed carbon nanotube networks act as electrodes and barium titanate–rubber interfaces act as triboelectric layers, were fabricated. Due to the reinforcing effects of the filler materials, the composites exhibit excellent mechanical properties, including high stretchability and toughness. These composites were evaluated for single-electrode-type TENG applications. Different triboelectric efficiencies suggest that TENG performance is strongly influenced by the optimum filler amounts and their dispersion behavior. It is evident that a rubber composite containing 2 phr CNTs and 50 phr barium titanate demonstrates better triboelectric efficiency than composites with higher CNT contents. Similarly, in composites with higher CNT content, increasing the amount of barium titanate leads to enhanced triboelectric effects. Although higher filler contents may sometimes result in increased triboelectric outputs, the associated enhancement in stiffness can negatively impact overall energy conversion efficiency. Due to dispersion challenges associated with polar barium titanate in a nonpolar rubber matrix, stearic acid was employed to improve filler dispersion. With improved filler dispersion, notable enhancements in both mechanical properties and triboelectric performance were observed. At an optimum amount of stearic acid, the composite exhibited increased toughness along with significant improvements in triboelectric efficiency. However, beyond the optimum stearic acid content, although mechanical properties may continue to improve due to better dispersion, the triboelectric efficiencies decline. This reduction is attributed to the presence of excess stearic acid-coated barium titanate particles that do not effectively participate in triboelectrification with the rubber matrix. Ultimately, the composite containing 50 phr barium titanate, 2 phr CNTs, and 1 phr stearic acid demonstrated the best performance, achieving power efficiency, power density, charge density, and charge efficiency values of 1.127 nW/N, 8.258 mW/m^3^, 0.146 nC/N, and 1.072 mC/m^3^, respectively, under a 2% cyclic strain at 0.85 Hz frequency. Although a threshold pressure or deformation is required to activate the TENG, the device operates more efficiently under lower pressures. Therefore, a thin composite sheet (0.02 × 6 × 5 cm^3^) was utilized for energy harvesting through hand patting, achieving significant power and charge densities of up to 26.04 W/m^3^ and 0.205 mC/m^3^, respectively, from a single hand pat. Due to its scalable energy generation efficiency, the composite also performed well in handwriting recognition, demonstrating high accuracy in time and sensitivity patterns. Therefore, this TENG composite shows strong potential for use in self-powered sensor devices with scalable energy generation capabilities.

## Figures and Tables

**Table 1:** Mixing compositions of different fillers and modifier per hundred grams of masterbatch rubber (phr).

Formulation	Amount of Masterbatch Rubber (g)	Amount of BaTiO_3_ (g)	Amount of CNTs (g)	Amount of Stearic Acid Modifier (g)
BT-50/CNT-1.5/	100	50	1.5	-
BT-50/CNT-2	100	50	2.0	-
BT-50/CNT-2.5/	100	50	2.5	-
BT-100/CNT-2.5	100	100	2.5	-
BT-50/CNT-2/SA-1	100	50	2	1
BT-50/CNT-2/SA-3	100	50	2	3
BT-50/CNT-2/SA-5	100	50	2	5

## Data Availability

The original contributions presented in this study are included in the article/Supplementary Material. Further inquiries can be directed to the corresponding authors.

## Supplementary Materials

The following supporting information can be downloaded at: https://www.mdpi.com/article/10.3390/polym17152016/s1, Figure S1: (a) Sheet and cylindrical rubber specimens, (b) diagram of energy harvesting setup, (c,d) instrumental setup with original sample. Figure S2: (a,b) Variation in compressive load with % of strain for different rubber composites. Figure S3: (a,b) Variation in tensile load with % of strain for different rubber composites. Figure S4: Changes in voltage and current from lowest to highest outputs for different rubber composites at 2% deformative loading–unloading cycle; (a,b) change in voltage and (c,d) change in current. Figure S5: Degradation in output voltage of BT-50/CNT-2/SA-1 composite with loading–unloading cyclic time; (a) 0 s–10 s, (b) 1000 s–1010 s, (c) 2000 s–2010 s, and (d) 3530 s–end of cycles.
