# Supplementary material for: High-Performance Barium Titanate, Carbon Nanotube, and Styrene–Butadiene Rubber-Based Single Composite TENG for Energy Harvesting and Handwriting Recognition
Source: Polymers (Basel). 2025 Jul 23;17(15):2016. doi: 10.3390/polym17152016 (PMC12349676; doi:10.3390/polym17152016)
Supplement: Supplementary file 1 [file polymers-17-02016-s001.zip › polymers-3702207-supplementary.pdf]

Supporting Information

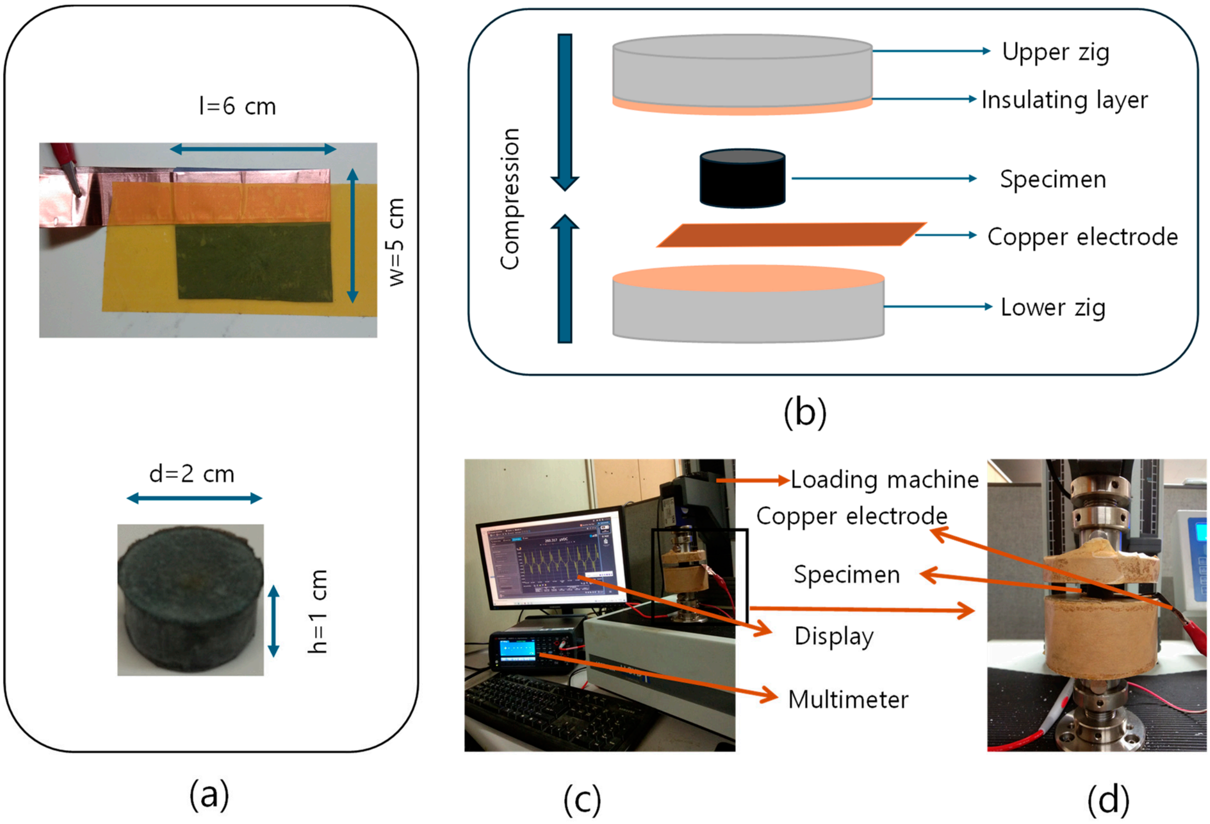

Figure S1. (a) sheet and cylindrical rubber specimens, (b) diagram of energy harvesting setup, (c-d) instrumental setup with original sample.

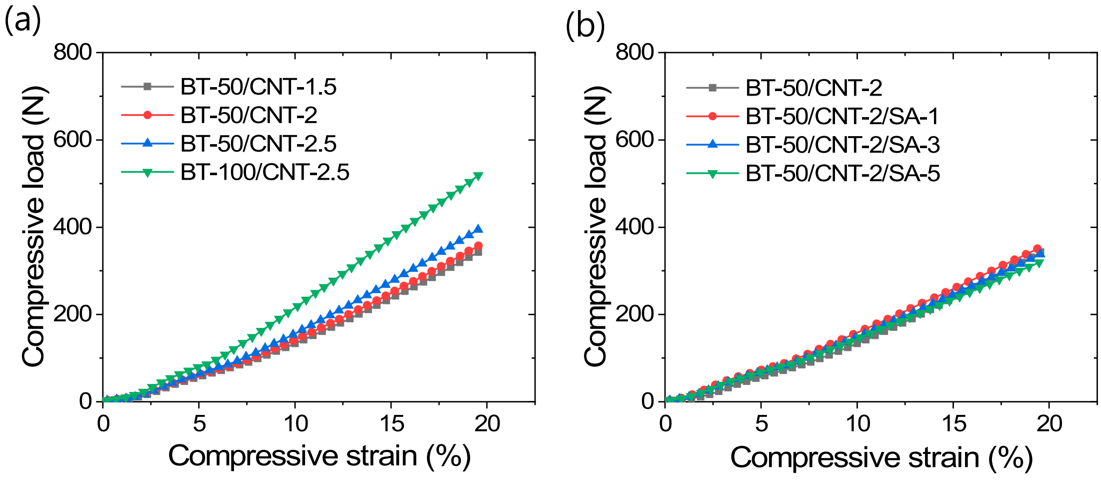

Figure S2. (a-b) Variation in compressive load with % of strain for different rubber composites.

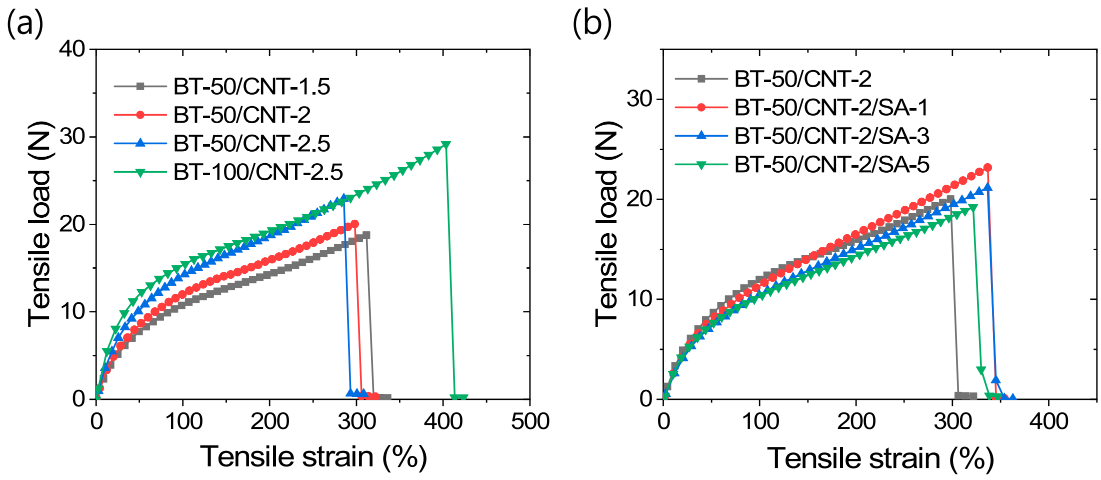

Figure S3. (a-b) Variation in tensile load with % of strain for different rubber composites.

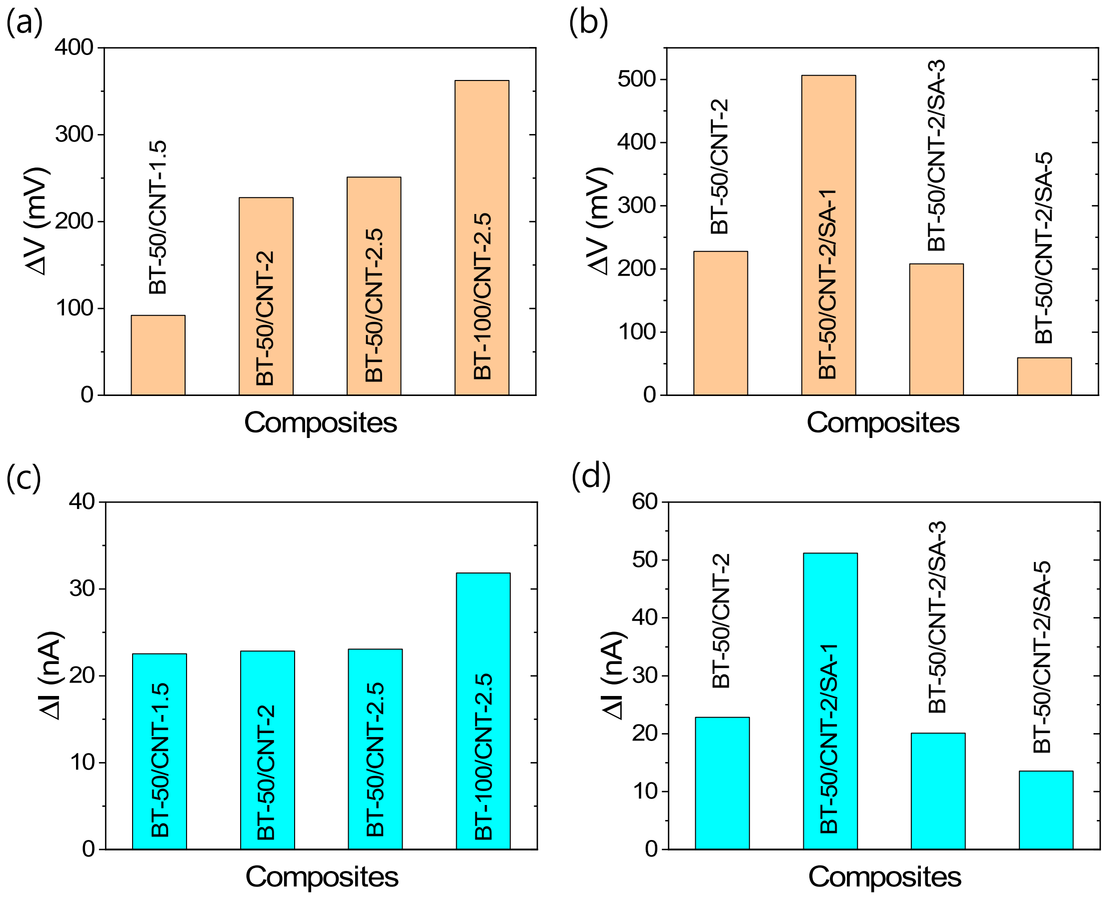

Figure S4. Changes in voltage and current from lowest to highest outputs for different rubber composites at 2% deformative loading-unloading cycle; (a-b) change in voltage and (c-d) change in current.

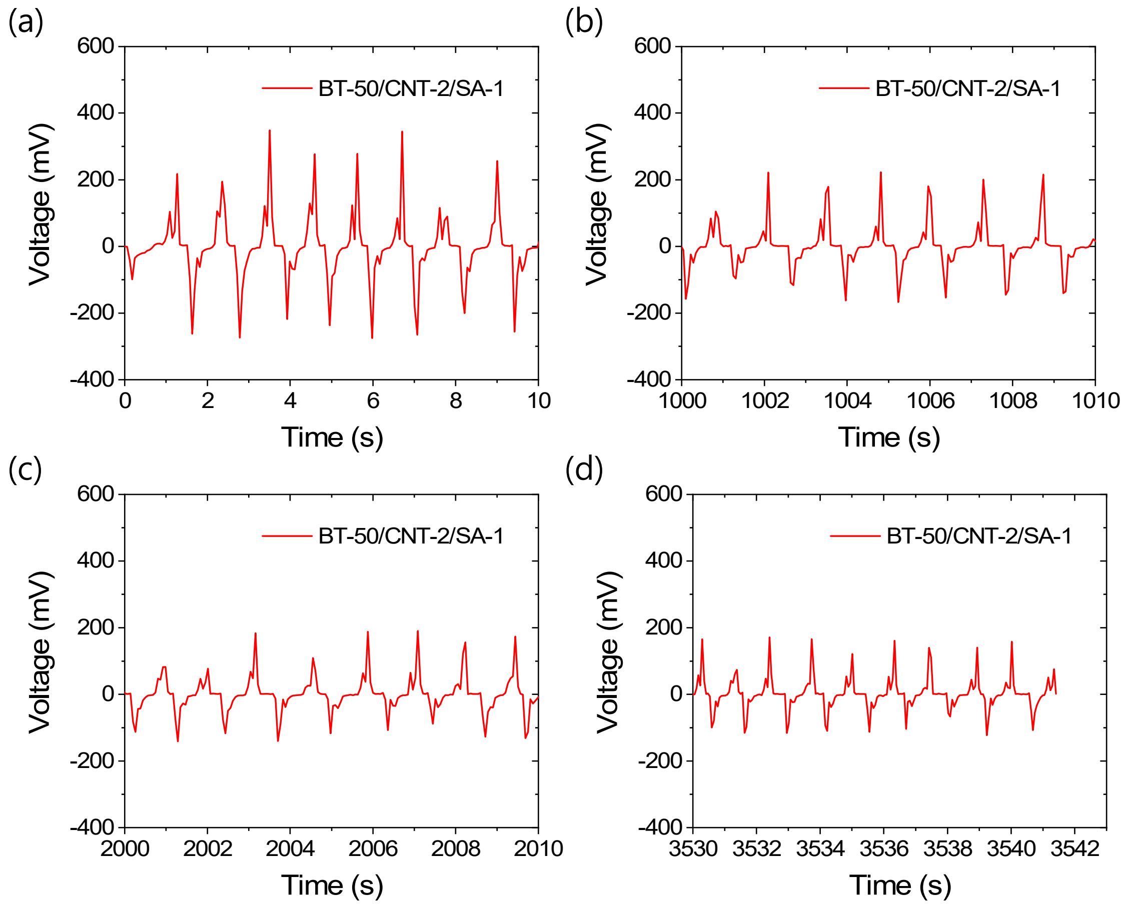

Figure S5. Figure 5S. Degradation in output voltage of BT-50/CNT-2/SA-1 composite with loading-unloading cyclic time; (a) 0s-10s (b) 1000s-1010s, (c) 2000s-2010s, and (d) 3530s-end of cycles.
